# The Effect of Row Structure on Soil Moisture Retrieval Accuracy from Passive Microwave Data

**DOI:** 10.1155/2014/964837

**Published:** 2014-10-15

**Authors:** Zheng Xingming, Zhao Kai, Li Yangyang, Ren Jianhua, Ding Yanling

**Affiliations:** ^1^Northeast Institute of Geographical and Agroecology, Chinese Academy of Sciences, Changchun 130102, China; ^2^Changchun Jingyuetan Remote Sensing Test Site, Chinese Academy of Sciences, Changchun 130102, China

## Abstract

Row structure causes the anisotropy of microwave brightness temperature (TB) of soil surface, and it also can affect soil moisture retrieval accuracy when its influence is ignored in the inversion model. To study the effect of typical row structure on the retrieved soil moisture and evaluate if there is a need to introduce this effect into the inversion model, two ground-based experiments were carried out in 2011. Based on the observed C-band TB, field soil and vegetation parameters, row structure rough surface assumption (*Q*_*p*_ model and discrete model), including the effect of row structure, and flat rough surface assumption (*Q*_*p*_ model), ignoring the effect of row structure, are used to model microwave TB of soil surface. Then, soil moisture can be retrieved, respectively, by minimizing the difference of the measured and modeled TB. The results show that soil moisture retrieval accuracy based on the row structure rough surface assumption is approximately 0.02 cm^3^/cm^3^ better than the flat rough surface assumption for vegetated soil, as well as 0.015 cm^3^/cm^3^ better for bare and wet soil. This result indicates that the effect of row structure cannot be ignored for accurately retrieving soil moisture of farmland surface when C-band is used.

## 1. Introduction

Land surface soil moisture not only affects vegetation growth, but also partly controls global water, energy, and carbon cycle, which is a key parameter for describing energy and water exchanges between land surface and atmosphere. Soil moisture is significant for hydrometeorology, ecological environment, and agricultural researches and is also an important data source for global water cycle, hydrological model of basin, crop growth and drought monitoring, and so forth [1–4].

Microwave remote sensing has become the primary source for large scale soil moisture estimation because of its strong sensitivity to soil moisture. Nevertheless, soil moisture from passive microwave remote sensing is affected by vegetation canopy, surface roughness, physical and chemical properties of soil, and atmospheric parameters. In addition, microwave emissivity of farmland soil surface is affected by surface row structure. For a better understanding of this effect on surface microwave brightness temperature, some physical models of modeling microwave brightness temperature of row structure surface are developed [5].

In this paper, discrete model is used to model microwave brightness temperature of row structure smooth soil surface. Before utilizing this model to simulate brightness temperature of row structure surface, we need to express row structure surface with a mathematical form. The results from [6] show that the* sine* function can better represent the height variation of row structure surface than the* trigonometric* function and the* trapezoidal* function. Also found in [6], the uncertainty from row structure could be ±0.03 in emissivity for L band as incidence angle of 10 degrees and ratio of row height to row spacing less than 0.2, which is considered negligible during soil moisture retrieval from passive microwave remote sensing. This conclusion is drawn in specific conditions, which may not be appropriate for other conditions, such as different incidence angles, different frequency, and different ratio of row height to row spacing.

Some researchers carry out studies of row structure surface with 95 cm and 105 cm row spacing [5–7] and obtain some conclusions about the effect of row structure on retrieval accuracy of soil moisture. For the typical row structure in northeast China, row spacing and row height are 65 cm and 15 cm, respectively. Can we ignore the effect of this typical row structure on brightness temperature when soil moisture is retrieved from passive microwave data?

The purpose of this paper is to reveal the effect of this specific row structure in northeast China on surface microwave brightness temperature and soil moisture retrieval accuracy.

## 2. Material and Method

### 2.1. Ground-Based Experiment

In 2011, two remote sensing experiments are carried out in Paoziyuan (125°7′11.7′′E, 43°45′32′′N), Changchun, China. The one was designed for analyzing the retrieval error of soil moisture caused by row structure. The other aims to determine whether the effect of row structure on soil moisture existed under vegetated soil.

#### 2.1.1. Experiment of Bare Soil

**Figure 1:**
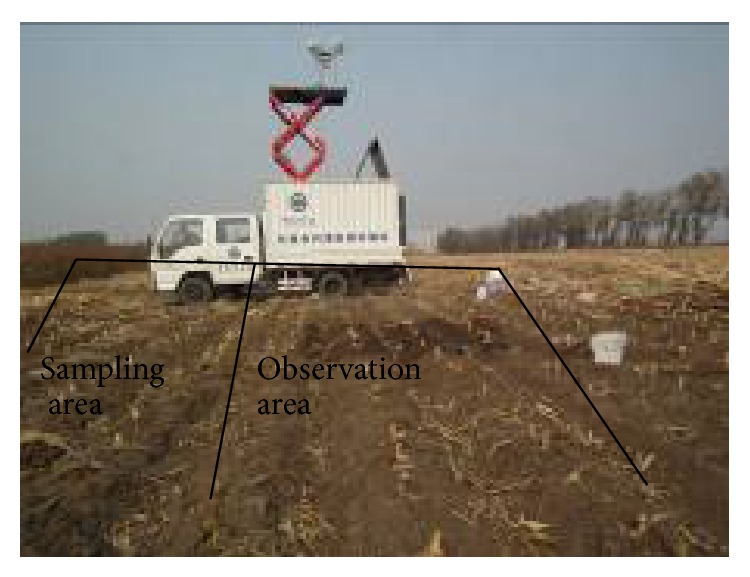
Truck-mounted C-band radiometer and its observing area.

**Figure 2:**
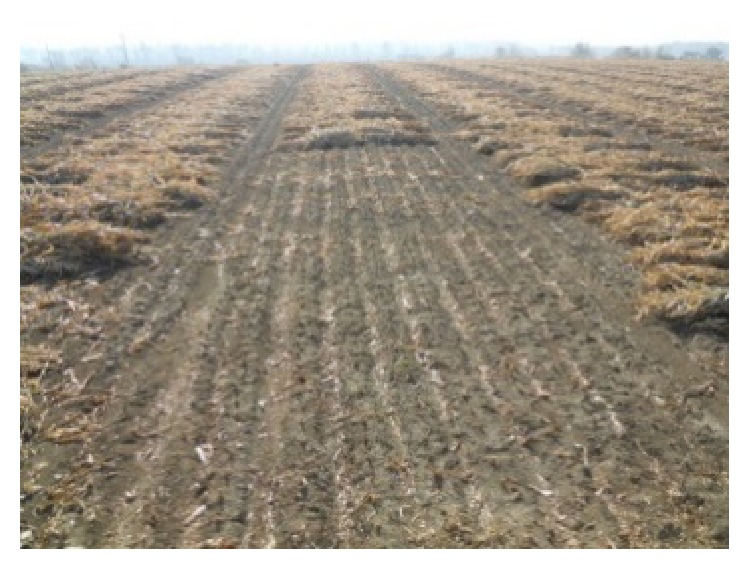
Soil surface after removing corn stalks.

In October 2011, surface microwave brightness temperature of bare soil is measured by truck-mounted C-band radiometer designed by Microwave Remote Sensing Group, Northeast Institute of Geography and Agroecology (IGA), Chinese Academy of Sciences (CAS) (Figure 1). The antenna of C-band radiometer used here has the main beam of 15°, and its height from ground is 5.8 m. At 50° incidence angle, the IFOV of the antenna is approximately ellipse with the semiaxis of 3.8 m and 1.8 m. The observation direction of C-band radiometer is parallel to the direction of row structure, so the azimuth angle is 0°. In the experimental area, row spacing and row height are 65 cm and 11 cm, respectively. Corn stalk randomly distributed in the soil surface needs to be removed from our experimental area to avoid its effect on surface microwave brightness temperature (Figure 2).

**Figure 3:**
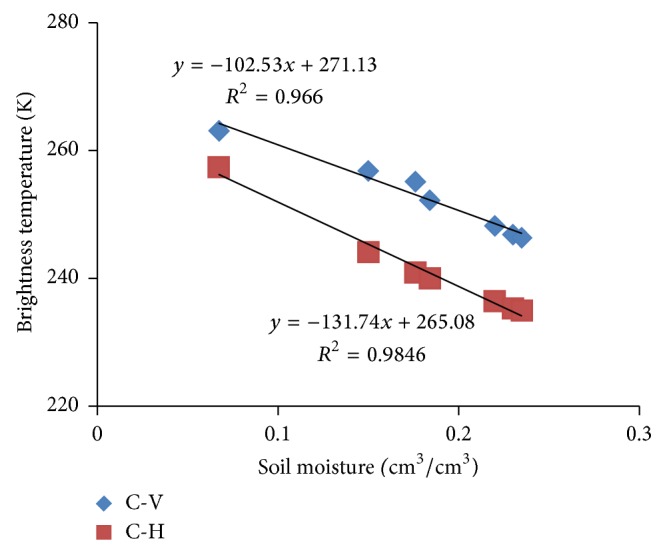
The change in C-band brightness temperature at both H and V polarization with field soil moisture at bare soil test area.

To reveal the effect of row structure in northeast China on the accuracy of retrieval soil moisture, we need to work under different soil moisture conditions. To obtain these data in a relatively short time, soil moisture within the antenna IFOV is changed by artificial watering method. By this method, bare soil was observed by ground-based radiometer at 7 different soil moisture (6 watering). The measured C-band brightness temperature at both polarizations decreases when farmland soil becomes wet (Figure 3).

At the same time of microwave measurement, 5 soil samples of 5 cm depth were collected by ring-knife and used to calculate soil parameters, such as soil moisture and soil bulk density. Soil texture can also be achieved from these soil samples through laboratory measurement. Soil temperature of 5 cm depth and 50 cm depth is measured by probe thermometer. Surface roughness is measured by 2 m length profiler with 4 repeats.

#### 2.1.2. Experiment of Vegetated Soil

The location of vegetation experiment area is the same as bare soil. Microwave brightness temperature of vegetated soil is measured from June 21, 2011, to September 21, 2011. The purpose of this experiment is to analyze the effect of row structure on microwave brightness temperature of vegetated soil.

**Figure 4:**
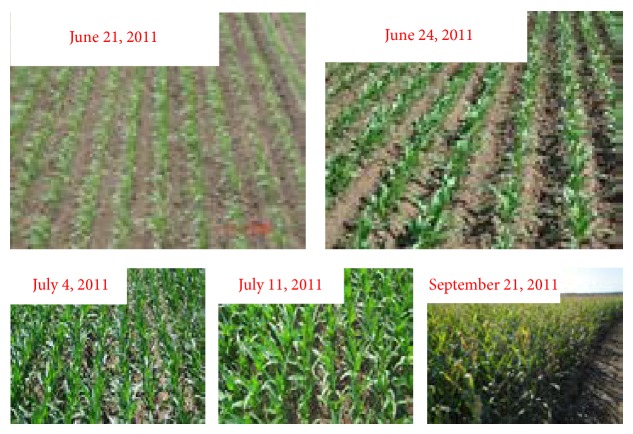
Corn canopy in different growth stages.

Six measurements are carried out on June 21, June 24, July 4, July 11, July 18, and September 21, and the corresponding DOY (date of year) is 201, 204, 214, 221, 228, and 291. The change of corn canopy during this experiment is shown in Figure 4, and no picture is photographed in July 18, 2011. From the beginning to the end of the experiment, corn canopy experiences six stages (seeding, jointing, big trumpet, tasseling, silking, and mature).

C-band radiometer is also used to measure microwave brightness temperature of vegetated soil. Unlike the bare soil experiment, the brightness temperature of vegetated soil at both H and V polarization is measured at incidence angle of 40°, 50°, 55°, 60°, and 70°. The measurement mode of microwave brightness temperature in vegetated soil is the same as bare soil. Also, the method for measuring soil parameter is the same as bare soil. To analyze the effect of corn canopy on microwave brightness temperature received by the C-band radiometer, vegetation water content (VWC) is sampled using the destructive method.

**Figure 5:**
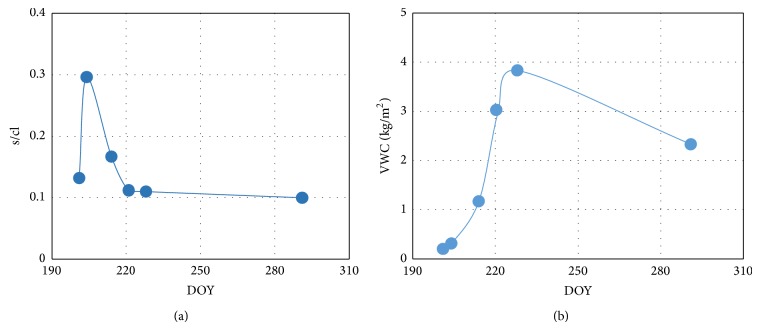
The change in s/cl (a) and VWC (b) with the sampling date. s/cl (the ratio of root mean square height and correlation length) denotes surface roughness parameter and VWC denotes vegetation water content.

Figure 5(a) shows the change of surface roughness with the sampling date in vegetated soil. The ratio of surface root mean square height and correlation length (s/cl) increases first and then decreases with the change of the sampling date. The maximum of s/cl appears at the day of 203 because soil surface is tilled. Then, due to the influence of rain, wind, and gravity, the surface roughness decreases until it becomes constant.

Vegetation water content (VWC) is commonly used to describe the effect of vegetation canopy on soil surface microwave radiation.  Figure 5(b) shows the change of vegetation water content with the sampling date. Also, vegetation water content increases first and then decreases with the sampling date. The maximum vegetation water content appears at the day of 228 because the height of corn canopy no longer increases and the moisture content in corn leaves becomes less after that day.

### 2.2. Microwave Model for Bare Soil and Vegetated Soil

#### 2.2.1. Bare Soil

Microwave brightness temperature of bare soil received by radiometer consists of soil and atmospheric contribution:

(1) $$\begin{array}{c} T_{p}\left(\theta ,\varphi \right)=\left(1-R_{p}\left(\theta ,\varphi \right)\right)T_{\text{Soil}}+\left(1+R_{p}\left(\theta ,\varphi \right)\right)T_{\text{atom}}, \end{array}$$

where *θ*, *φ*, and *p* represent incidence angle, azimuth angle, and polarization, respectively. Here, the value of *φ* is 0° because the observation direction of radiometer is always parallel to the direction of row structure. *R*_*p*_(*θ*, *φ*) represents reflectivity of rough soil surface.

Soil effective temperature (*T*_Soil_) can be expressed as the function of soil temperature and soil moisture at different soil depth. The approximate expression of soil effective temperature is *T*_Soil_ = *T*_Deep_ + *C*(*T*_Surf_ − *T*_Deep_); *T*_Deep_ and *T*_Surf_ are soil temperature at the depth of 50 cm and 0–5 cm, respectively. The *C* value is 0.667 ± 0.008 for C-band [8]. *T*_atom_ refers to downward atmospheric brightness temperature introduced in Section 2.2.3.

Affected by human activities, farmland surface could be approximately classed into two categories: the one is random rough surface, and the other is row structure rough surface. The row structure rough surface is the combination of random rough surface and row structure smooth surface. To model brightness temperature of this surface, two surface radiation models are used here. The one, named *Q*_*p*_ model, can model microwave brightness temperature of random rough surface, and the other, named discrete model, can model microwave brightness temperature of row structure smooth surface. Microwave brightness temperature of row structure rough surface can be modeled by combining *Q*_*p*_ model and discrete model.

*Discrete Model*. Three steps are required to model brightness temperature of row structure smooth surface in discrete model. Step 1: the surface within IFOV is discretized as the small area with a fixed spatial interval of Δ*x*, and Δ*x* is 1/30*P*_rs_ (*P*_rs_: row spacing) here. Step 2: microwave brightness temperature of the discretized small area is modeled based on the energy conservation law and the coordinate transformation. Step 3: microwave brightness temperature of soil surface within the IFOV is calculated from the modeled brightness temperature of small area and antenna pattern function. For the details of step 1 and step 2, please refer to [5].

In step 3, ignoring contributions from side lobe of radiometer, the* p*-polarized microwave brightness temperature *T*_*A*_(*θ*, *φ*, *p*) of soil surface at an incidence angle of *θ* and an azimuth angle of *φ* is given by

(2) $$\begin{array}{c} T_{A}\left(\theta ,\phi ,p\right)=\frac{\iint_{\Omega_{M}}T_{B}\left(\theta ,\phi ,p\right)F_{n}\left(\gamma \right)d\Omega_{s}}{\iint_{\Omega_{M}}F_{n}\left(\gamma \right)d\Omega_{s}}, \end{array}$$

where *T*_*B*_(*θ*, *φ*, *p*) is the brightness temperature of the small area *dΩ*_*s*_ observed by radiometer from the direction (*θ*, *φ*) and *Ω*_*s*_ is the antenna main beam solid angle. For simplicity, the antenna's normalized radiation pattern is assumed to be circularly symmetric; therefore, it is a function of the angle *γ* only. Usually, the central part of the antenna's main beam is approximately Gaussian in shape and can be fitted to

(3) $$\begin{array}{c} F_{n}\left(\gamma \right)=\exp\left(-\frac{a\gamma^{2}}{\beta^{2}}\right), \end{array}$$

where *β* is the* half-power beam-width* and *a* = 2.77. In such a case, the integration limits in (2) may be restricted to the range of *γ* between 0 and 2*β* without a noticeable loss of computational accuracy. Combining (2) and (3), the *p*-polarized microwave brightness temperature *T*_*A*_(*θ*, *φ*, *p*) of row structure smooth surface is modeled. Then, the reflectivity of row structure smooth surface *r*_*p*_(*θ*, *φ*) can be expressed as

(4) $$\begin{array}{c} r_{p}\left(\theta ,\varphi \right)=1-e_{p}\left(\theta ,\varphi \right)=1-\frac{T_{A}\left(\theta ,\varphi ,p\right)}{T_{\text{Soil}}}, \end{array}$$

where *p* denotes H or V polarization and *e*_*p*_(*θ*, *φ*) is the emissivity of row structure smooth surface at incidence angle of (*θ*, *φ*).

*Q*_*p*_ *Model*. *Q*_*p*_ model, developed based on advanced integrated equation model (AIEM) [9], is used to calculate the reflectivity of soil surface *R*_*p*_(*θ*, *φ*), and its expression is as follows:

(5) $$\begin{array}{c} R_{p}\left(\theta ,\varphi \right)=Q_{p}\cdot r_{p}\left(\theta ,\varphi \right)+\left(1-Q_{p}\right)\cdot r_{q}\left(\theta ,\varphi \right), \end{array}$$

where *Q*_*p*_ is polarization coupling coefficient in *p* polarization and can be expressed as a function of surface roughness (surface* root mean square* height and correlation length). When row structure exists in the soil surface, *r*_*p*,*q*_(*θ*, *φ*) denotes the reflectivity of row structure smooth soil surface in *p* or *q* polarization, which can be computed from (4). When no row structure exists in the soil surface, *r*_*p*,*q*_(*θ*, *φ*) is equal to the Fresnel reflectivity of flat smooth soil surface [5]. In this paper, soil complex dielectric constant is computed based on Generalized Refractive Mixing Dielectric Model (GRMD) [10]. Then, microwave brightness temperature of soil surface can be computed by combining (1) and (5) if the downward atmospheric microwave brightness temperature is known.

For bare soil, two surface assumptions are used in this paper. One of them is flat rough surface assumption, and it assumes that farmland surface is flat and rough, and *Q*_*p*_ model is used to model farmland surface microwave brightness temperature. The other assumption considers farmland surface as row structure rough surface and both discrete model and *Q*_*p*_ model are required to model farmland surface microwave brightness temperature. Comparing their retrieval accuracy of two assumptions, a better assumption is determined for modeling farmland surface brightness temperature.

#### 2.2.2. Vegetated Soil

Microwave brightness temperature radiated from vegetated soil received by radiometer consists of soil, vegetation, and atmospheric contribution:

(6) TBP=(1−ω)(1+γpRp)TV+(1−Rp)γpTSoil +(1+γp2Rp)Tatom,

where *T*_*V*_ and *T*_Soil_ are corn canopy temperature and soil effective temperature, respectively. *R*_*p*_ is the reflectivity of soil surface introduced above. To illustrate the effect of row structure on soil moisture retrieval accuracy, two assumptions are also used as bare soil experiment, and two methods are used to calculate *R*_*p*_: (1) the one method is the combination of discrete model and *Q*_*p*_ model, and the effect of row structure is considered for calculating farmland surface reflectivity, (2) the other method is using *Q*_*p*_ model alone, and farmland surface reflectivity is computed based on flat rough surface assumption, ignoring row structure effect.

In (6), *γ* and *ω* denote the transmissivity and single scattering albedo of vegetation canopy, respectively. The value of single scattering albedo in C-band (6.6 GHz) is 0.096 ± 0.01 from the results of [11], and this value is used in this paper. The transmissivity of corn canopy (*γ*_*p*_) in *p* polarization can be expressed as

(7) $$\begin{array}{c} \gamma_{p}=e^{-\tau \text{sec}\theta }, \end{array}$$

where *θ* is incidence angle and *τ* is vegetation optical thickness, which can be expressed as the function of vegetation water content (VWC): *τ* = *b* × VWC. Parameter *b* is the empirical coefficient and a value of the parameter *b* of 0.15 is representative of most agricultural crops, with the exception of grasses [12], and this value of 0.15 is also used in this paper.

#### 2.2.3. Atmospheric Microwave Radiation

Although microwave has the ability to penetrate clouds, atmosphere is not completely transparent and has absorption and radiation processes. *T*_atom_ under clear sky conditions is calculated with radiation transfer model described in [13]. The result shows *T*_atom_ at 6.925 GHz and V polarization is up to 4 K. Because it is cloudy during the experiment, *T*_atom_ cannot be ignored. The expression of *T*_atom_ is [13]

(8) $$\begin{array}{c} T_{\text{atom}}\left(\theta \right)=\left(1-e^{-\tau_{d}(\theta )}\right)T_{a}, \end{array}$$

where *T*_*a*_ is atmospheric equivalent temperature and can be expressed as

(9) $$\begin{array}{c} T_{a}=\frac{\int_{0}^{H}T\left(z\right)d\exp\left(-\tau_{d}\left(\theta \right)\right)}{\int_{0}^{H}d\exp\left(-\tau_{d}\left(\theta \right)\right)}, \end{array}$$

where *τ*_*d*_(*θ*) is atmospheric optical thickness in the incidence angle of *θ* and *τ*_*d*_(*θ*) = *τ*_NAD_/cos⁡(*θ*) and *τ*_NAD_ is the optical thickness at nadir, which can be expressed as the integral of extinction coefficient including absorption and scattering coefficient of water vapor and oxygen. Atmospheric parameters for calculating nadir optical thickness are provided by the Department of Atmospheric Sciences, University of Wyoming (http://www.weather.uwyo.edu/upperair/sounding.html).

## 3. Result

### 3.1. Bare Soil

**Figure 6:**
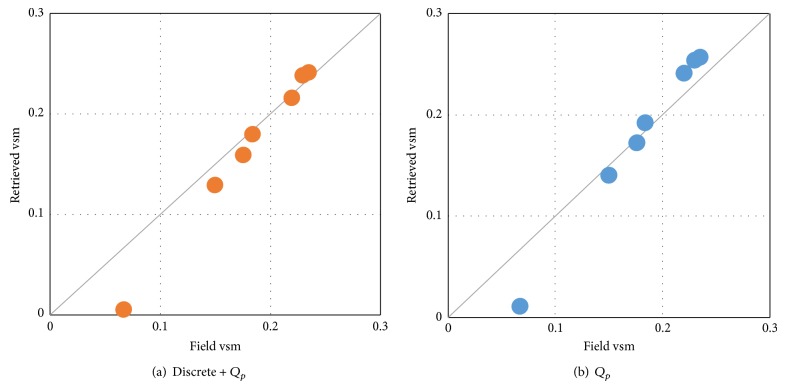
Field and retrieved volumetric soil moisture (cm^3^/cm^3^).

Based on the measured multiangle C-band brightness temperature and the sampling soil and vegetation parameters, soil moisture can be retrieved by integrating discrete model and *Q*_*p*_ model using nonlinear least square method. The absolute error of retrieval soil moisture is 0.017 cm^3^/cm^3^ for row structure rough surface assumption. The absolute error of retrieval soil moisture is 0.021 cm^3^/cm^3^ for flat rough surface assumption (Figure 6).

This result reveals that the assumption which introduces row structure effect into surface microwave radiation model can improve farmland soil moisture retrieval accuracy. As shown in Table 1, soil moisture inversion error for row structure rough surface assumption is slightly greater than flat rough surface assumption, and the difference between them is approximately 0.005 cm^3^/cm^3^ when soil is dry. Soil moisture inversion error for row structure rough surface assumption is lower than flat rough surface assumption, and the difference is approximately 0.015 cm^3^/cm^3^ when soil is wet. This is because drier soil has high surface emissivity and low surface reflectivity, and the reflected radiation by row structure for dry soil is smaller than wet soil with high surface reflectivity. Soil surface reflectivity becomes greater with the increasing soil moisture, thus the reflected radiation by row structure also becomes greater. Thus, row structure rough surface assumption including row structure effect is important for improving soil moisture retrieval accuracy, especially for wet soil.

### 3.2. Vegetated Soil

**Figure 7:**
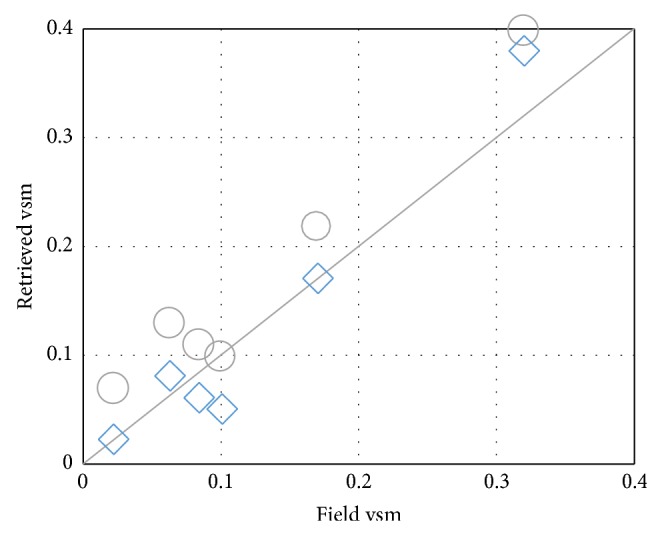
The scatter plot between field and retrieved volumetric soil moisture (vsm). (◯: *Q*_*p*_ model, ◊: discrete model and *Q*_*p*_ model, and —: 1 : 1 line).

Based on multiangle (40°, 50°, 55°, 60°, and 70°) C-band brightness temperature in vegetated soil, soil moisture is retrieved based on two different farmland surface assumptions. Compared with field volumetric soil moisture, the* rmse* of retrieval soil moisture based on row structure rough surface assumption (discrete model and *Q*_*p*_ model) is 0.0253 cm^3^/cm^3^, and the* rmse *of retrieval soil moisture for flat rough surface assumption (*Q*_*p*_ model) is 0.046 cm^3^/cm^3^. The* rmse* of retrieval soil moisture based on row structure rough surface assumption is 0.021 cm^3^/cm^3^ lower than that of flat rough surface assumption. Also found from Figure 7, the retrieval volumetric soil moisture based on row structure surface assumption is closer to 1 : 1 line than the flat surface assumption. This result indicates that row structure surface assumption can improve the accuracy of retrieval soil moisture even if vegetation exists on the soil surface.

## 4. Discussions

How does row structure affect surface microwave brightness temperature? Compared with the flat rough soil surface, microwave radiation of row structure rough surface has two distinctions: (1) the assumption that soil surface microwave brightness temperature is isotropic is no longer valid; (2) the emissivity of row structure rough surface is different with flat rough surface even if other conditions are the same. The reason of these differences is that directionality of row structure causes the asymmetry of farmland surface structure. Due to the presence of row structure, soil moisture changes with the height of soil surface. The result is that positive terrain (ridge) has lower soil moisture than negative terrain (furrow) due to the effect of gravity and shadow.

## 5. Conclusion

For explaining the effect of row structure on soil moisture retrieval accuracy from passive microwave brightness temperature, taking ground-based soil and vegetation parameters as the input dataset, surface soil moisture is retrieved by nonlinear least squares method from C-band and multiangle microwave brightness temperature based on the row structure rough surface assumption (discrete model and *Q*_*p*_ model) and flat rough surface assumption (*Q*_*p*_ model). Some conclusions are found by comparing field and retrieved soil moisture. (1) Soil moisture retrieved from the row structure rough surface assumption has better accuracy than the flat rough surface assumption. (2) The effect of row structure in soil moisture retrieval still exists when soil surface is covered by corn canopy. This is proved by the fact that soil moisture retrieval error of the row structure rough surface assumption is approximately 0.021 cm^3^/cm^3^ lower than that of the flat rough surface assumption in vegetated soil and 0.015 cm^3^/cm^3^for bare and wet soil.Thus, high precision of soil moisture retrieval from passive microwave remote sensing needs to introduce row structure effect into rough surface radiation model.

## Figures and Tables

**Table 1:** Retrieved volumetric soil moisture and its error.

Watering time	Field vsm	Discrete + *Q*_*p*_		*Q*_*p*_	
		vsm	Error	vsm	Error
0	0.067	0.006	0.061	0.011	0.056
1	0.150	0.129	0.021	0.140	0.010
2	0.176	0.160	0.016	0.172	0.004
3	0.184	0.179	0.005	0.192	−0.008
4	0.220	0.216	0.004	0.241	−0.021
5	0.230	0.238	−0.008	0.253	−0.023
6	0.235	0.241	−0.006	0.256	−0.021
